# A new method for 2D gel spot alignment: application to the analysis of large sample sets in clinical proteomics

**DOI:** 10.1186/1471-2105-9-460

**Published:** 2008-10-28

**Authors:** Sabine Pérès, Laurence Molina, Nicolas Salvetat, Claude Granier, Franck Molina

**Affiliations:** 1 Sysdiag CNRS FRE 3009 BIO-RAD. Cap delta/Parc Euromédecine, 1682 rue de la Valsière, CS 61003, 34184 MONTPELLIER Cedex 4, France

## Abstract

**Background:**

In current comparative proteomics studies, the large number of images generated by 2D gels is currently compared using spot matching algorithms. Unfortunately, differences in gel migration and sample variability make efficient spot alignment very difficult to obtain, and, as consequence most of the software alignments return noisy gel matching which needs to be manually adjusted by the user.

**Results:**

We present Sili2DGel an algorithm for automatic spot alignment that uses data from recursive gel matching and returns meaningful Spot Alignment Positions (SAP) for a given set of gels. In the algorithm, the data are represented by a graph and SAP by specific subgraphs. The results are returned under various forms (clickable synthetic gel, text file, etc.). We have applied Sili2DGel to study the variability of the urinary proteome from 20 healthy subjects.

**Conclusion:**

Sili2DGel performs noiseless automatic spot alignment for variability studies (as well as classical differential expression studies) of biological samples. It is very useful for typical clinical proteomic studies with large number of experiments.

## Background

Two-dimensional gel electrophoresis is a high resolution technique that is widely used in proteomics to separate thousands of proteins from a complex sample. After separation, a 2D map is obtained in which each protein, or isoform, is represented by a spot. In clinical proteomics the user has to analyze 2D maps of a large number of proteins as, very often, dozens of controls and pathological samples are compared. To allow this comparison, maps from all gels have to be aligned. Unfortunately, differences in gel migration and sample variability can render spot alignment very difficult [1]. There are two types of general limitations for 2D profiling: i) those due to variations in proteome composition and ii) those due to inadequacy of the analytical methods [2]. Computer-aided image analysis contributes to the second kind of limitations and may lead to analytical pitfalls [1]. For instance, 2D gel migration can cause geometrical distortion and variable spot coordinates in different gels [3,4] for many reasons [5]. During the process of image analysis, spot alignment is a critical step since it will condition spot comparison. Spot alignment can be performed mainly in two way: i) spot detection followed by spot-based image warping and finally spot alignment, or ii) pixel-based image warping followed by spot detection and then spot alignment [3]. In the first method the spot-based image warping corrects image distortion using user-defined landmark spots. This process can eventually be fully automated by making the spatial correction implicit [3]. The spot alignment is often expressed using a fusion gel that is representative to the whole experiment [6,7]. When the number of gels to be aligned is high, the distortion has to be modelled with a low-order polynomial transformation [3,8]. In this case, local geometric distortions are poorly corrected leading to an increase of noise in the spot alignment. In the second (pixel-based warping) method, the spatial correction is performed directly from raw-image data, taking advantage of techniques originating from image processing research. This approach leads to a more flexible image distortion (followed by spot detection) virtually eliminating matching problems. However, even if this method is more convenient, it remains bias due for instance to discontinuous change in intensity among the set of aligned gels [9] ending to affected spot intensity quantitation. In addition, the user must systematically adjust or control the spot alignment process by hand [4,6]. This is time consuming and a source of errors.

**Figure 1:**
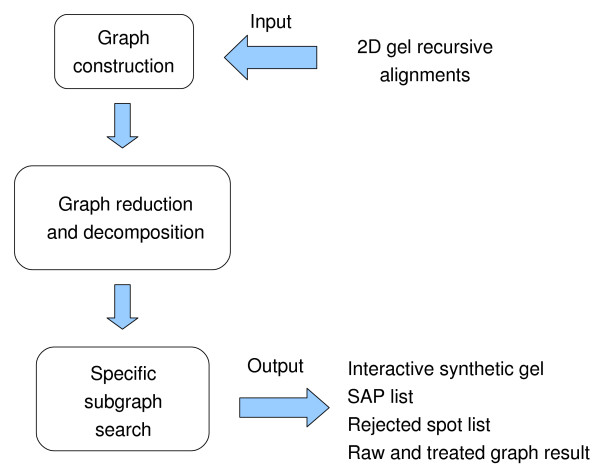
**Principle of the Sili2DGel algorithm**. Sili2DGel represents the result of recursive gel matching with a graph, decomposes it in disconnected subgraphs, searches specific subgraphs which represent the SAP and returns them under various formats.

Up to now comparative analysis of 2D gels has been based on the utilization of commercial gel analysis systems (e.g. Pdquest [10], Melanie [11], Samespots [12], Proteomweaver, Gellab [13], etc.), which identify spots of interest by image comparison, a process called gel matching. While some systems pair each gel of a matching set against a single "reference gel" (e.g. Melanie, Pdquest, etc.), some other algorithms follow the concept of recursive gel matching (e.g. Samespot, Proteomweaver, etc.). This means that each gel of a matching set is recursively used as "reference gel" once during the matching process. However, the resulting spot alignment remains noisy and is not suitable for further statistical analysis. We propose herein a new algorithm for automatic spot alignment, called Sili2DGel, which uses data from recursive gel matching to return only the meaningful Spot Alignment Positions (SAP) for a given set of gels (Figure 1). Sili2DGel is based on graph theory, input data are represented by a graph in which specific subgraphs are searched. The results are returned under various formats (clickable synthetic gel, text file, etc.). This approach provides the user with an automatic and efficient spot alignment tool suitable for analysis of a large set of 2D gels.

## Implementation

### Alignment representation using graph theory

**Figure 2:**
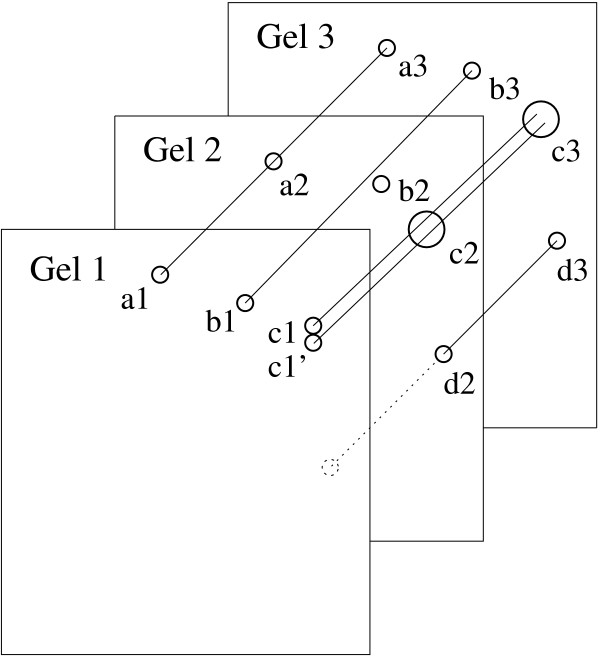
**Example of alignments of three gels**. Spot *a*_1 _matches with *a*_2 _and *a*_3_, thus *A*(*a*_1_) = {*a*_1_; *a*_2_; *a*_3_}, *A*(*b*_1_) = {*b*_1_; *b*_3_}; similarly *A*(*c*_1_) = {*c*_1_; *c*_2_; *c*_3_} and *A*(${c^{\prime }}_{1}$) = {${c^{\prime }}_{1}$; *c*_2_; *c*_3_}.

Ideally, after different experiments, a given protein should be represented by spots displayed at the same coordinates on each gel. However, if only a single reference gel is selected for a match set, spots that are not found in that reference gel will not form alignments. For instance in Figure 2, if Gel 1 is the reference gel then the alignment of {d2;d3} will not be recorded as they are not present in Gel 1. Moreover, different kinds of distortions can skew the matching. As a consequence, some spots are likely to end up in an alignment where they should not, and others will not be attributed to an alignment when they should. For instance in Figure 2, assuming that the spots *b*_1_, *b*_2 _and *b*_3 _represent the same protein, if *b*_2 _matches with *b*_3_, it should be aligned with *b*_1_. Spots which belong to an alignment due to an error have to be eliminated (*noise spots*), and spots which are missing have to be restored (*missing spots*). The meaningful Spot Alignment Positions (SAP) correspond to the set of spots which represent the same protein after exclusion of the noise spots and reinstatement of the missing spots. SAP can be determined by analysing the alignments given by the recursive gel matching method. One should note that this program depends on prior accurate processing of the spots indentifications and the preliminary spot alignments which are not trivial tasks. So a spot that has not been recognised due to low signal level in spot identifications of the prior process, will be missing in the following analysis.

If *N *is the number of gels and *S *the set of all spots of the *N *gels, then for any spot *i*, gel matching will give all the spots *j *which match with *i*. We use the notation *i *→ *j *when spot *i *matches with spot *j*. An alignment of spot *i *includes *i *and all its matching spots (see Figure 2), noted as A(*i*):

*A*(*i*) = {*j *∈ *S *: *i *→ *j*} ∪ {*i*}.

Alignments are represented by a weighted undirected graph which is called *matching graph*. A node corresponds to a specific spot of a given gel and an edge represents the matches between spots. The weight of an edge is the number of matches between two spots. Therefore, it is less or equal to the number of gels.

If *G*_*N *_is the matching graph of *N *gels and |*S*| the size of a set *S*, then we have:

*G*_*N *_= (*V*, *E*, *w*)

where :

• *V *= *S *is the set of vertices of *G*_*N*_

• *E *= {(*i*, *j*) : *i *∈ *S*, *j *∈ *A*(*i*), *i *≠ *j*} is the set of edges of *G*_*N*_

• *w*(*i*, *j*) = |{*k *∈ *S *: *i *∈ *A*(*k*), *j *∈ *A*(*k*)}|, ∀(*i*, *j*) ∈ *E *is the weight of the edge (*i*, *j*).

**Figure 3:**
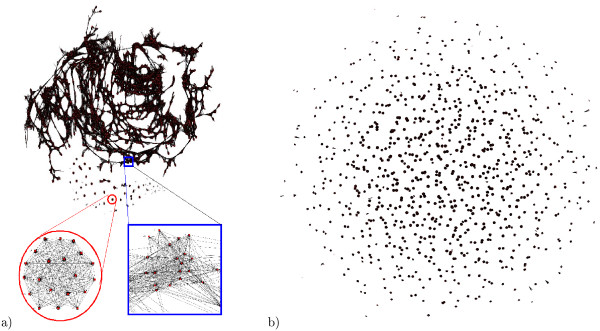
**Graph representations**. (a) raw graph (before treatment) and (b) treated graph (after treatment). Graphs were represented with the Tulip software [24]. The raw graph is composed of good SAP (3a, bottom left panel) and noisy SAP (3a, bottom right panel)

Nodes are labelled with the name of the gel and the number of the spot. Edges are labelled with their weight. SAP are represented in the graph by high density zones, *i.e*. zones where a lot of nodes are pairwise adjacent (Figure 3a bottom left panel). Most of the time, there are many associations that make the graph highly incorrectly connected (Figure 3a top panel). It is therefore necessary to clean the graph to find the sets of nodes which represent the same spots. This is done by removing the edges which represent wrong connexions.

The search of SAP on *N *gels comes down to finding specific subgraphs of the matching graph *G*_*N*_. In the best case, all the spots, which represent the same element, will pairwise match in *n *gels, with *n *≤ *N*. In the matching graph the nodes representing these *n *spots are all connected together. This subgraph is called a *clique *(*i.e*. a complete graph); moreover all its edges are weighted by *n*. A graph *G *= (*V*, *E*) is a clique if all vertices are pairwise adjacent, *i.e*. ∀*i*, *j *∈ *V *with *i *≠ *j*, we have (*i*, *j*) ∈ *E*. However, alignments are not always perfect. The case where all the spots match at least once, but are not all pairwise adjacent, is represented in the matching graph by a clique in which at least one edge is weighted by a value lower than n. In the worst case, some spots will be missing in an alignment even through they should belong to it. If two spots from different gels never match together during the whole recursive matching procedure, but match with many of the other spots, they are not adjacents in the matching graph and the subgraph is not a clique. This subgraph contains a clique and some other nodes which are adjacents to several nodes of the clique; we call it a *pseudoclique*. In all cases, SAP are represented by dense clusters of nodes in the graph (*i.e*. nodes that are highly connected to each other) which are either cliques or pseudocliques.

### Algorithm of SAP identification

#### Reducing the graph

Finding a maximal clique is a classical NP-hard problem [14], thus exact algorithms are guaranteed to return a solution only in a time which increases exponentially with the number of vertices in the graph [15]. Therefore, one can expect exact solution methods to have limited performance on large datasets. To overcome this difficulty, we decomposed the graph into reduced subgraphs and then we determined the SAP in the corresponding reduced search spaces.

To reduce the search space, the graph is partitioned in all its connected components (*i.e *the maximal connected subgraphs). Before searching the connected component, we suppressed the edges weighting 1, as we assumed that an edge with a weight of 1 was not sufficient to belong to a pseudoclique, and that it could not represent an alignment of size 2 (which contains 2 spots). The suppression of these edges will not distort the results because if these spots are really in the same alignment, they should have a high connectivity with some other spots of the alignment and so they would be later restored.

Pseudocliques represent very dense clusters of the graph. To select very dense clusters, the *isthmuses *(*i.e*. the edges which separate a set of nodes of the graph in two highly connected subgraphs) have to be eliminated. The strength metric [16] allows the isthmuses determination by measuring how much an edge is likely to separate a graph in two highly connected subgraphs. It is defined as:

$$sm(u,v)=\frac{\left|W_{uv}\right|}{\left|W_{uv}\right|+\left|M_{u}\right|+\left|M_{v}\right|}+\frac{e\left(M_{u},W_{uv}\right)+e\left(M_{v},W_{uv}\right)+e\left(M_{u},M_{v}\right)+e\left(W_{uv}\right)}{\left|M_{u}\right|\cdot \left|W_{uv}\right|+\left|M_{v}\right|\cdot \left|W_{uv}\right|+\left|M_{u}\right|\cdot \left|M_{v}\right|+\left(\begin{array}{c} \left|W_{uv}\right|\\ 2 \end{array}\right)}$$

Where *u*, *v *∈ *V*, *M*_*u *_= *N*_*u*_\*N*_*v *_and *W*_*uv *_= *N*_*u *_∩ *N*_*v *_with *N*(*u*), *N*(*v*) denote the neighborhoods of *u *and *v*. *e*(*A*, *B*) (or *e*(*A*)) denotes the number of edges between the two sets *A *and *B *(or within a set *A*). The first term counts the number of triads (cycles of length 3) containing the edge (*u*, *v*) and the later computes the relative number of cycles of size 4 containing the edge.

Values of *sm *are between 0 and 2. A low value indicates that the edge is more likely to act as an isthmus whereas a high value signifies that it is potentially at the centre of a cluster. It is worth noting that a null strength metric is an isthmus and a value of two is an edge which belongs to an isolated clique. Thus, edges with a small strength metric (lower than a threshold value *sm*) are suppressed to reduce the graph. If the graph is highly connected, *sm *value should not to be too low to allow a good reduction of the graph. Then, the connected components are calculated and SAP can be researched into all these reduced graphs. Table 1 represents the overall algorithm of SAP search and Table 2 the algorithm of cluster search for each connected component of the graph.

**Table 1:** Algorithm of SAP search

SAP_search(*sm*, *γ*):	
1. Graph construction.	• Remove edges having a weight of 1.

2. Graph reduction:	• Remove edges for which the strength metric is lower than *sm*.
	• Decompose the graph into its connected components.

3. For each connected components, cluster_search(*γ*).	

4. Return the new graph.	

**Table 2:** Algorithm of clusters search

Clusters_search(*γ*):	
1. Clique and pseudoclique search:	• Find the maximal cliques and remove the cliques of size two if their nodes have more than 1 neighbour
	• For all cliques *C*, remove the nodes *n *for which max *w*(*n*, *i*)_*i*∈*C *_≤ *τ*(*C*)
	• For all cliques *C*, add to *C *the *γ*-dense nodes *n *such that min *w*(*n*, *i*)_*i*∈*C *_≥ *τ*(*C*)
	• Remove from the list of cluster the included clusters.

2. Select clusters according to their *s*-value:	• Remove the "worst clusters": For all clusters *C*_1_, *C*_2 _such that |$V_{c_{1}}$| ≥ |$V_{c_{2}}$|, if |$V_{c_{1}}$ ∩ $V_{c_{2}}$| ≥ *γ *× |$V_{c_{1}}$| and *s*(*C*_1_) ≥ *s*(*C*_2_) and *Gelnb*(*C*_1_) ≥ *Gelnb*(*C*_2_) then remove *C*_2_.
	• For all clusters *C*_1_, *C*_2 _such that |$V_{c_{1}}$ ∩ $V_{c_{2}}$| ≥ *γ *× *min*(|$V_{c_{1}}$|, |$V_{c_{2}}$|), add the nodes of *min*(*s*(*C*_1_), *s*(*C*_2_)) which have a maximal weight greater than *τ*(*C*_*min*_) in *max*(*s*(*C*_1_), *s*(*C*_2_)).

3. Select nodes which belong to several clusters:	• For all nodes *n *which belong to several clusters, remove *n *from all clusters where it does not have its maximal *MeanW*.

#### Clusters search

The principle of the algorithm of cluster search is to find sets of nodes which are highly connected but not necessary all pairwise adjacent and with edges of high weight value (with respect to the size of the set). Maximal cliques were searched by using the Bron-Kerbosch algorithm [17] with the heuristics of Koch [18] and Cazals [19]. Moreover, we kept the cliques of size 2 only if they were disconnected from the rest of the graph. The search of cliques did not take into account the weight of the edges, which had to be checked; moreover the nodes which are highly connected to the clique had to be added. After finding all the maximal cliques, we assumed that the nodes characterized by edges with a small maximal weight in the clique were noisy spots and therefore we removed them. If they are not, they will be restored in the next steps. In conclusion, the nodes *n *which have a maximal weight in a clique *C *smaller than a threshold value *τ*(*C*) (1) are removed (*i.e*. if ∃*n *∈ *C s.t *max *w*(*n*, *i*)_*i*∈*C *_≤ *τ*(*C*) then *n *is removed from *C*). On the principle, the threshold value insures a high tolerance to weakly connected nodes within a great clique and a low tolerance within a small clique.

If *G*_*N *_= (*V*, *E*) is a matching graph of *N *gels and *C *= (*V*_*c*_, *E*_*c*_) a subgraph of *G*_*N*_, then we define the threshold of *C *as a function *τ *such that:

(1) $$\tau (C)=\left|V_{c}\right|\times \frac{N}{N+GelNb(C)},$$

where *GelNb*(*C*) is the number of gels in *C*. We can note that *GelNb*(*C*) can be different of |*V*_*c*_| because all nodes in *C *are not always coming from different gels, (c.f. spots *c*_1 _and ${c^{\prime }}_{1}$ in Figure 2). Thus, *GelNb*(*C*) ≤ |*V*_*c*_|. *τ*(*C*) gets its values from interval [$\frac{1}{2}$|*V*_*c*_|, |*V*_*c*_|]. The threshold gives a value which is close to $\frac{1}{2}$|*V*_*c*_| if the clique is of great size and a value closed to |*V*_*c*_| if the clique is of small size. It is worth noting that this formula is valid for clusters of nodes which are not cliques.

A node *n *is selected to be added to a clique *C *if it is connected with at least *γ *× |*V*_*c*_| nodes of *C *where *γ *∈ [0, 1] is a parameter. *γ *is the percentage of nodes that a node has to be connected to belong to a clique. This value is chosen depending the quality of the gels and/or the matching. If qualities are not very good, *γ *has to be low to tolerate nodes which might have been missed in the matching process else has to *γ *be high not to give noisy SAP. We say that *n *is *γ*-dense in *C*. If *G*_*N *_= (*V*, *E*) is a a graph and *C *= (*V*_*c*_, *E*_*c*_) a subgraph of *G*_*N*_, then a node *i *∈ *V *is *γ*-dense in *C *if there is a subset ${V^{\prime }}_{c}$ ⊂ *V*_*c *_such that for each *j *∈ ${V^{\prime }}_{c}$ the edge (*i*, *j*) ∈ *E *and |${V^{\prime }}_{c}$| ≥ *γ *× |*V*_*c*_|. Moreover a node which is added to a clique *C *must have matched several times with the nodes of *C *(*i.e *min *w*(*n*, *i*)_*i*∈*C *_≥ *τ*(*C*)). If at least one node has been added to a clique, the resulting set of nodes is not a clique anymore; we will called it in the following a cluster. It is worth noting that all the *γ*-dense nodes of a maximal clique *C *of a graph *G*_*N *_belong to a maximal clique of *G*_*N*_. This means that, at this step, many clusters are likely to be included in other clusters. When this happens, the included clusters are removed.

#### Select clusters according to their quality criteria value

Clusters which share a lot of nodes can remain, whereas, clusters which are characterized by a small size and low quality criteria will be removed. The clustering quality measure [20] for a cluster *C*, *s*(*C*), is defined as follows:

(2) $$s(C)=\frac{\left|E_{c}\right|}{\left(\begin{array}{c} \left|V_{c}\right|\\ 2 \end{array}\right)},$$

Where the binomial coefficient $\left(\begin{array}{c} \left|V_{c}\right|\\ 2 \end{array}\right)$ gives the maximum number of edges between the vertices in *C*. *s*(*C*) is the ratio between the number of edges and the highest possible number of edges. For all clusters *C *in *G*, we have *s*(*C*) ∈ [0, 1]. If *s*(*C*) = 1, *C *is a clique. Thus, a cluster represents a good alignment if its *s*-value is close to 1. If we find two clusters such that $\left|V_{c_{1}}\cap V_{c_{2}}\right|\geq \gamma \times max\left(\left|V_{c_{1}}\right|,\left|V_{c_{2}}\right|\right)$ we will remove the one with the lower *s*-value and the lower number of gels. As few clusters of small size have been removed, so clusters which share a lot of nodes with a bigger one may still remain. Our aim is to adjust the clusters in order to obtain a high value of *s*(*C*). Therefore, if there are two clusters such that the cluster with smaller number of nodes is *γ*-dense in the other (*i.e *$\left|V_{c_{1}}\cap V_{c_{2}}\right|\geq \gamma \times min\left(\left|V_{c_{1}}\right|,\left|V_{c_{2}}\right|\right)$), the nodes of the cluster with smaller *s*-value which have a maximal weight greater than *τ*(*C*_*min*_) are added in the cluster with greater *s*-value.

#### Select nodes which belong to several clusters

The last step is to remove nodes, which belong to several clusters, from their worst clusters. If *G*_*N *_= (*V*, *E*) is a graph and *C *= (*V*_*c*_, *E*_*c*_) a subgraph of *G*_*N*_, then the mean weight of a node *n *∈ *V *in *C *is defined as the sum of all weights of all edges of *n *divided by the total number of vertices in *C*:

(3) $$MeanW(n,C)=\frac{\sum_{i\in C}w(n,i)}{\left|V_{c}\right|}.$$

Thus, if a node *n *belongs to several clusters, *n *remains only in the cluster where *n *has its highest mean weight.

## Results and discussion

We used Sili2DGel to study the variability of the urinary proteome from 20 healthy subjects. After 2D gel electrophoresis, silver straining and imaging as in [21], a recursive matching was performed with the Melanie software to identify every spots in each gel. The matching graph of these alignments had 16 386 nodes and 236 593 edges.

**Figure 4:**
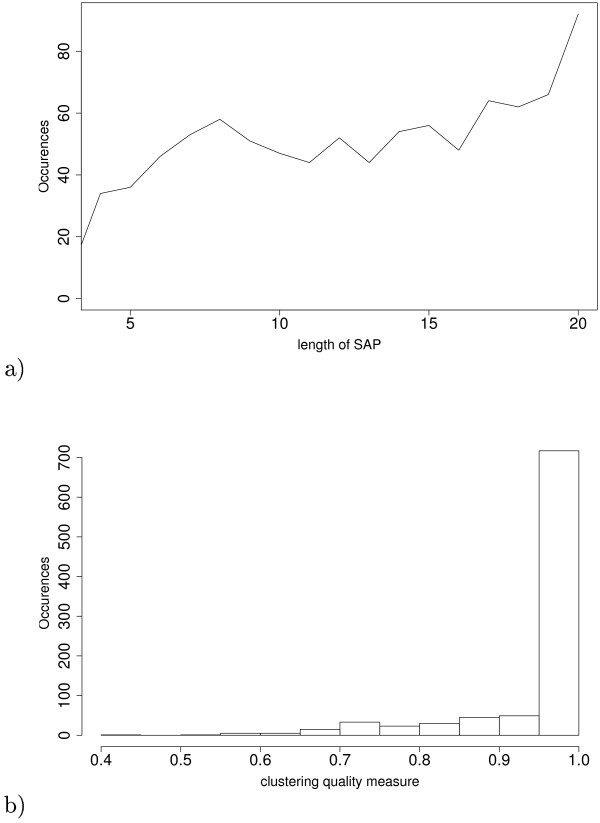
**SAP distribution and clustering measure**. (a) SAP distribution *i.e*. frequencies of the number of gels in different SAP and (b) clustering measure distribution

The raw matching graph (Figure 3a) allowed us to notice that the spots (*i*.*e *nodes of the graph) were very connected while the graph should have been composed of subgraphs which look like cliques. Therefore, it was not possible to make a relevant large scale statistical analysis at that stage. By applying Sili2DGel which withdrew background noises with parameter settings *γ *= 0.4 and *sm *= 0.8, we obtained 924 SAP of which 634 were cliques, 152 contained several spots in the same gels, 92 were conserved in all gels (Figure 4a) and only 25 had a clustering measure lower than 0.7 (Figure 4b). The closer *sm *is to zero, the more an edge will represent an isthmus and the closer *sm *is to 2, the more the edge in the centre of a clique. Looking at the raw data, we never found any edges representing an isthmus. So, after probing various values for the *sm *parameter, we found a value of 0.8 as the best compromise. The resulting graph contained 11 746 nodes and 80 769 edges (Figure 3b). All the subgraphs were clusters which represented the alignments and were either cliques or pseudocliques. These clusters represented good choices because the *s*-value for 770 of them was greater than 0.9 (Figure 4b) and only few clusters with a low *s*-value were left. Our software provided a synthetic gel that conveniently represented the SAP distribution and spot conservation among the studied gels (Figure 4a). We observed that spot conservation in the urinary proteome was heterogeneous. Indeed, by looking at the SAP length distribution we observed occurrences for all the possible SAP length from 3 to 20 gels. Interestingly, we noticed that the highest occurrence is found for the spots strictly conserved among the 20 gels. The more variable spots (SAP length of 4) are more rare in this study. This heterogeneity is consistent with experimental data found in the literature [22,23].

**Figure 5:**
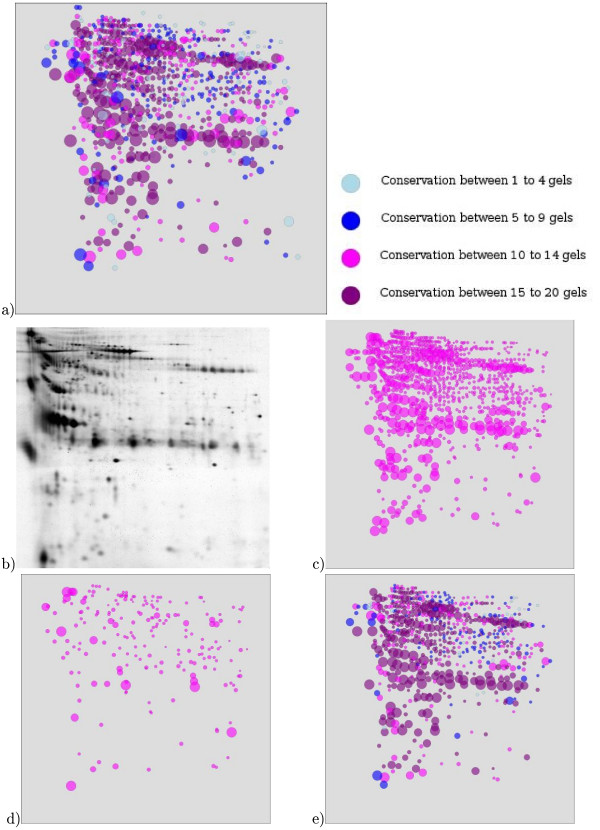
**Synthetic gels**. (a) SAP of the synthetic gel, (b) Gel1 image, (c) raw spot list from Gel1, (d) rejected spots from Gel1 and (e) SAP-related spots of Gel1.

**Figure 6:**
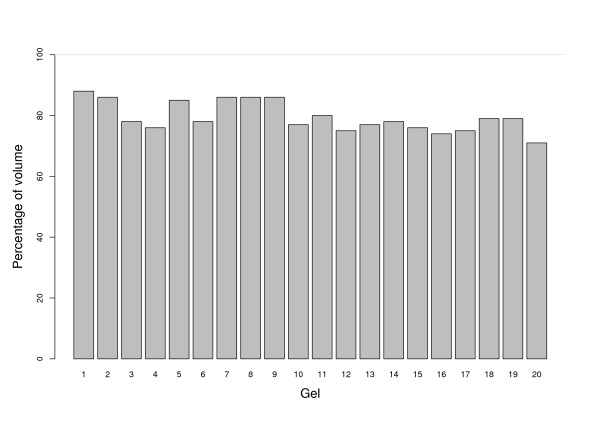
**Percentage of volume of SAP-related spots from the synthetic gel**. Signal before treatment is considered at 100%. One can note that 80% of spot intensity is conserved.

The analysis showed that 152 SAP contained several spots from the same gels. This suggests that for a given SAP, gels where single spots are found have a lower resolution than gels with duplicated spots in the corresponding SAP (for instance, see spots *c*_2 _and *c*_3 _of Figure 2). As a consequence, the resolution of a specific spot of a low resolution gel could be enhanced by using the corresponding spot from a better resolution gel. This set of heterogeneous SAP is provided to the user to allow specific analysis. Our software provided a synthetic gel which corresponds to all the SAP found among all the gels which have been identified (Figure 5a) with the algorithm. Figure 5 shows the raw spots from Gel1 before edge reduction (Figure 5c) built from the Gel 1 image (Figure 5b) and the difference between the SAP-related spots from Gel 1 (Figure 5d) and the rejected spots (Figure 5e). When we compared the percentage of the volume of the SAP-related spots in the synthetic gel to that of the rejected spots for Gel 1, we observed an average conservation of 80% (Figure 6) of the original signal. Indeed, out of the 947 spots of Gel1 (100% of volume), 717 spots (88% of volume) were related to a SAP of the synthetic gel. The rejected spots, which represented on average the remaining 12% of the spots, were considered as ambiguous signals (see rejected spots set for Gel1 in figure 5d).

We also calculated the overall signal loss after Sili2Dgel spot alignment by comparing the total volume of each gel before and after treatment (Figure 6). All together, the sum of the percentage of the conserved intensity of all SAP-related spots (1598) in the synthetic gel represented 80% of the sum of the percentage of intensity of all spots (2000) from the original gels. The 924 SAP of the synthetic gel covered 80% of the experimental signal. The remaining 20% of the signal was found in the set of rejected spots after spot alignment, and could be accessed by the user in the output table for possible further manual analysis (see also synthetic gel in Figure 5d). The SAP-related spots constituted the set of data that were considered suitable for further statistical analysis.

## Conclusion

In comparative proteomics studies, the large number of images generated by 2D gels is currently compared using spot matching algorithms. However, most of the software alignments return noisy gel matching which needs to be manually adjusted by the biologist. Moreover, several of these systems pair each gel only against a single reference gel and therefore some spots might be missed. To restore them, it is necessary to make recursive alignments. To meet the needs of clinical proteomics of comparing large sets of 2D gels, we have developed Sili2Dgel an automatic gel alignment method based on graph theory to find SAP (without manual adjustment) after a recursive alignment procedure. This method first constructs a matching graph and then reduces its complexity by searching all its maximal cliques, adding the *γ*-dense nodes with a high minimal weight, selecting the clusters with high size and quality values and selecting nodes which belong to several clusters. Each cluster is considered as a SAP in the synthetic gel and indicates the equivalent spot position in the complete set of gels.

All SAP-related spots are available to the user for further statistical analysis. In addition, our method allows one to address recurrent clinical questions about the variability of biological samples leading to the issue of the conservation of proteins in the studied proteome. We used Sili2Dgel to analyze 20 normal urinary proteomes and we could show that spot conservation was heterogeneous, probably reflecting individual variations.

Finally, the input and output files of Sili2DGel (tabular text files) are compatible with the main 2D gel analysis systems on the market and this allows users to easily combine our method with their familiar environment, making Sili2Dgel a companion tool for users of current commercial proteome analysis software. It performs, after recursive gel matching, an automatic global spot alignment of large sets of related gels with little loss of global signal and a large number of SAP. If needed, the SAP can be used to enhance the resolution of other spots using the spot resolution from the best gels of the set. Sili2DGel performs noiseless automatic spot alignment for variability studies (as well as classical differential expression studies) of biological samples. It makes it very useful for typical clinical proteomic studies with large number of experiments.

## Availability and requirements

• Project name: Sili2DGel

• Project home page: 

• Operating system(s): Platform independent

• Programming language: Java

## Authors' contributions

SP conceived and designed the software. LM performed the gels separation analyses and the recursive gel matching. FM and CG conceived and coordinated the study. NS participated in the development of the software. All authors participated in development of the methods and preparation of the manuscript. All authors read and approved the final manuscript.
